# Risk factors for lower renal compensation after nephrectomy: an analysis of living kidney donors in an Amazonian cohort

**DOI:** 10.1590/2175-8239-JBN-2023-0134en

**Published:** 2024-02-09

**Authors:** Luan Moraes Ferreira, Gisela Gomes Batista, Leoneide Érica Maduro Bouillet, Emanuel Pinheiro Esposito

**Affiliations:** 1 Universidade do Estado do Pará, Faculdade de Medicina, Santarém, PA, Brazil.

**Keywords:** Kidney Transplantation, Living Donors, Obesity, Albuminuria, Proteinuria

## Abstract

**Introduction:**

Living donor kidney transplantation is considered the ideal renal replacement therapy because it has a lower complication rate and allows an efficient response to the high demand for grafts in the healthcare system. Careful selection and adequate monitoring of donors is a key element in transplantation. Individuals at greater risk of developing kidney dysfunction after nephrectomy must be identified.

**Objective:**

To identify risk factors associated with a renal compensation rate (CR) below 70% 12 months after nephrectomy.

**Methods:**

This observational retrospective longitudinal study included living kidney donors followed up at the Lower Amazon Regional Hospital between 2016 and 2022. Data related to sociodemographic variables, comorbid conditions and kidney function parameters were collected.

**Results:**

The study enrolled 32 patients. Fourteen (43.75%) had a CR < 70% 12 months after kidney donation. Logistic regression found obesity (Odds Ratio [95%CI]: 10.6 [1.7–65.2]), albuminuria (Odds Ratio [95%CI]: 2.41 [1.2–4.84]) and proteinuria (Odds Ratio [95%CI]: 1.14 [1.03–1.25]) as risk factors. Glomerular filtration rate was a protective factor (Odds Ratio [95% CI]: 0.92 [0.85–0.99]).

**Conclusion:**

Obesity, albuminuria and proteinuria adversely affected short-term renal compensation rate. Further studies are needed to uncover the prognostic implications tied to these risk factors. Our findings also supported the need for careful individualized assessment of potential donors and closer monitoring of individuals at higher risk.

## Introduction

Chronic kidney disease (CKD) is one of the main causes of death in modern society. It is an important public health issue and a condition that affects more than 10% of the world’s population, with approximately 840 million cases worldwide^1^. It is defined by the presence of structural abnormalities, decreased kidney function and/or kidney injury for a period of more than three months. Severity of involvement is rated based on glomerular filtration rate (GFR) and albuminuria. Individuals with end-stage renal disease (GFR < 15 mL/min/1.73 m^2^) require renal replacement therapy (RRT)^2^. RRT includes a wide range of therapies, among which living donor transplant is considered ideal, since it presents lower graft loss rates and longer survival when compared to deceased donor transplant, in addition to imposing significantly fewer limitations in the ability to perform activities of daily living daily when compared to other therapies such as peritoneal dialysis or hemodialysis^3,4^.

According to the World Health Organization (WHO) and the Brazilian Organ Transplant Association (ABTO), Brazil was the fourth largest kidney transplant center in absolute numbers in the world, with 5,306 procedures performed in 2022, in a list topped by the United States, China and India. Approximately 14% of these transplants (n = 733) were performed with grafts from living donors, with trends showing growth in future years^5,6^.

After nephrectomy, donors lose approximately 50% of their kidney mass, which invariably leads to decreases in the GFR in the post-transplant period^7^. The remaining kidney is expected to compensate for this loss via glomerular hyperfiltration, causing a recovery to approximately 70% of the baseline GFR after one year^8,9,10^.

Although the risk of post-nephrectomy renal dysfunction was initially deemed non-significant, based on the underlying idea that the selection of healthy donors would produce risk levels lower than those of the general population, studies with better group matching demonstrated a small, but statistically significant, long-term risk of end-stage renal disease in this population^11,12,13^.

Recent research has looked into donor short-term kidney function. A study carried out in 2018 in Spain found statistically significant differences in renal compensation rates among living kidney donors after one year of follow-up^7^. In an analysis carried out in 2020 in South Korea, obesity and the body mass index (BMI) were associated with more pronounced decreases in post-nephrectomy GFR^14^. However, current literature lacks evidence regarding the factors that might influence kidney function recovery in the short term after donation and which might help to identify individuals at greater risk of developing CKD.

One of the central goals of living kidney donor selection processes is the mitigation of risks to donors. Multiple steps to ensure safety and ample information are provided to donors, and the scientific literature on the factors associated with worse short- and long-term post-donation prognosis is constantly updated to expand the safety net around potential donors and allow the individualized monitoring of individuals at greater risk^15^. This study aimed to identify risk factors associated with renal compensation rates < 70% one year after nephrectomy.

## Methods

This observational retrospective longitudinal study included a cohort of patients of both sexes and different ages who underwent nephrectomy for kidney donation at a tertiary hospital located in the Central Amazon region between January 2016 and December 2022. Patients who did not attend outpatient follow-up visits 12 months after kidney transplantation or who had undergone a nephrectomy within less than a year of the date of data collection were excluded.

**Figure 1.**
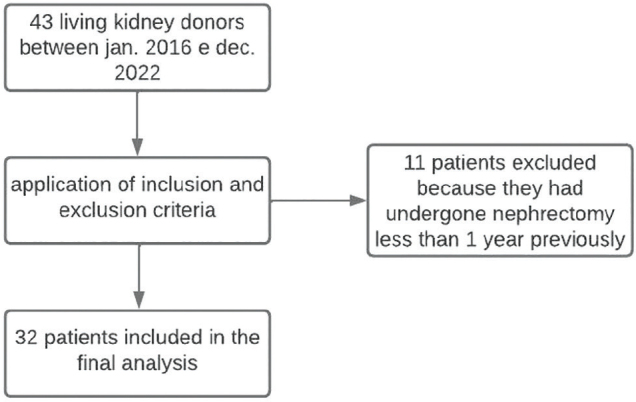
Flowchart of the application inclusion and exclusion criteria.

Figure 1 illustrates the inclusion and exclusion criteria used to include patients in the study population.

The following demographic variables were analyzed: sex, age and race/color. The following variables of clinical interest were analyzed: comorbid conditions (obesity, defined as having a BMI ≥ 30 kg/m^2^, as recommended by the National Institute for Health and Care Excellence; systemic arterial hypertension; dyslipidemia; alcoholism; and smoking), blood pressure and kidney function parameters (creatinine; GFR; albuminuria; and proteinuria)^16^. Measurements of albuminuria and proteinuria were obtained from 24-hour urine collection. The CKD-EPI 2021^17^ formula was used to estimate the GFR. Serum creatinine levels in the last pre-donation outpatient visit were taken as a reference to estimate pre-transplant GFR. Creatinine levels at the follow-up visit one year after nephrectomy were used to calculate the GFR 12 months after donation. Our study did not take endogenous creatinine clearance into account, since this parameter is not considered in the tests routinely performed for kidney donors at the chosen center.

Data from the medical records of the included patients were collected from April 1 to 15, 2023. The collected data included information from the last pre-nephrectomy visit and the outpatient follow-up visit held one year after donation. The renal compensation rate was calculated based on the GFR 12 months after nephrectomy and the pre-donation GFR, as described by Burballa et al.^7^: (Renal compensation rate): (GFR 1 year after nephrectomy/pre-nephrectomy GFR)*100.

In accordance with the literature on living kidney donors, the cutoff point from which renal compensation rate 12 months after nephrectomy was deemed normal was set at 70%^7–10^.

For purposes of descriptive analysis, absolute and relative frequencies were used for categorical variables; means and standard deviations for quantitative variables with a normal distribution; and medians and interquartile ranges for variables with non-normal distributions. The normality of the sample was tested using the Shapiro-Wilk test. The chi-square test was used in comparisons between categorical variables, while the T-test was used to compare between numerical variables. Possible risk factors for a compensation rate < 70% were also checked using logistic regression, odds ratios, and a 95% confidence interval (CI). Statistical analysis was performed on software package Stata version 14.0. Differences with a p < 0.05 were considered statistically significant.

This study was carried out in accordance with Resolution 466/2012 of the National Health Council and was approved by the Research Ethics Committee of the State University of Pará (certificate no. 67526923.0.0000.5168/ Opinion Number: 5.719.668). All study participants signed an informed consent form.

## Results

Thirty-two participants were included in the final analysis. There was a predominance of male individuals (n = 17, 53.2%) and persons of mixed race/color (n = 29, 90.6%); participant mean age was 40.9 ± 8.8 years. Regarding clinical parameters, the predominant comorbidity was obesity (n = 10, 31.2%). Mean systolic and diastolic blood pressure seen in study population were 119.6 (± 9.6) and 76.7 (± 5.4), respectively; mean blood pressure was 90.9 (± 6.1). In terms of kidney function, we observed an increase in creatinine levels over the first 12 months after nephrectomy (0.86 ± 0.14 to 1.2 ± 0.24) and a decrease in the GFR (104 ± 12.3 to 69.8 ± 16.8). In 12 months, albuminuria and proteinuria increased from 8.5 ± 5.5 mg to 10.8 ± 5 mg and from 58.3 ± 31.9 mg to 79.5 ± 33,7 mg, respectively. The study population’s pre-donation sociodemographic, clinical and kidney function parameters 12 months are described in Table 1.

**Table 1.** Sociodemographic and clinical characterisation of living kidney donors in a tertiary hospital in the amazon from 2016 to 2022, brazil

Variable	Frequency
Gender (n, %)	
***Male***	***17 (53.2)***
***Female***	***15 (46,8)***
Age (mean, ±SD)	***40.9 (± 8.8)***
Race (n, %)	
***White***	***2 (6.2)***
***Brown***	***29 (90.6)***
***Black***	***1 (3.1)***
Co-morbities (n, %)	
***Obesity***	***10 (31.2)***
***High blood pressure***	***3 (9.3)***
***Dyslipidemia***	***1 (3.1)***
***Tobacco use***	***5 (15.6)***
***Alcoholism***	***5 (15.6)***
Arterial blood pressure (mean, ± SD)	
***Systolic pressure***	***119.6 (± 9.6)***
***Diastolic pressure***	***76.7 (± 5.4)***
***Mean arterial pressure***	***90.9 (± 6.1)***
Pre-nephrectomy renal function (mean, ± SD)	
***Creatinine mg/dL***	***0.86 (± 0.14)***
***GFR (CKD-EPI 2021)***	***104 (± 12.3)***
***Albuminuria mg/24 hours***	***8.5 (± 5.5)***
***Proteinuria mg/24 hours***	***58.3 (± 31.9)***
Renal function after 12 months (mean, ± SD)	
***Creatinine mg/dL***	***1.2 (± 0.24)***
***GFR (CKD-EPI 2021)***	***69.8 (± 16.8)***
***Albuminuria mg/24 hours***	***10.8 (± 5)***
***Proteinuria mg/24 hours***	***79.5 (± 33.7)***
***Renal function CR*** (%, ±)	***69.5% (± 10.3)***
TOTAL (n)	32

SD: standard deviation. CR: compensation rate. GFR: glomerular filtration rate. Source: The authors, 2023.

The 32 individuals included in the study were divided into two groups based on renal compensation rate one year after nephrectomy. Group α featured patients with a compensation rate < 70% and Group β included individuals with a compensation > 70%. Statistically significant differences were observed in the following pre-donation variables: obesity, GFR, albuminuria and proteinuria. All kidney function variables were statistically different 12 months after nephrectomy. The comparisons between groups are described in Table 2.

**Table 2:** Difference between groups regarding the renal function compensation rate 1 year after nephrectomy in a tertiary hospital in the amazon, 2016 to 2022, brazil

Variable	Total		
	Group α – renal function CR < 70%	Grupo β – renal function CR > 70%	*p* value
Gender (n, %)	*** ***	*** ***	*** ***
***Male***	***7 (50)***	***10 (55.5)***	***0.95***
***Female***	***7 (50)***	***8 (44.4)***	
Age (mean, ± SD)	***42.5 (± 9.2)***	***39.7 (± 8.4)***	***0.18***
Race (n, %)	*** ***	*** ***	*** ***
***White***	***1 (7.1)***	***1 (5.5)***	***0.98***
***Brown***	***12 (85.7)***	***17 (94.4)***	
***Black***	***1 (7.1)***	***0***	
Co-morbities (n, %)	*** ***	*** ***	*** ***
***Obesity***	***8 (57.1)***	***2 (11.1)***	***0.005***
***High blood presure***	***2 (14.2)***	***1 (5.5)***	***0.4***
***Dyslipidemia***	***1 (4.1)***	***0***	***N.A.***
***Tobacco use***	***3 (21.4)***	***2 (11.1)***	***0.3***
***Alcoholism***	***3 (21.4)***	***2 (11.1)***	***0.3***
Arterial blood pressure (mean, ± SD)	*** ***	*** ***	*** ***
***Systolic pressure***	***120.7 (± 9.9)***	***118 (± 9.6)***	***0.3***
***Diastolic pressure***	***78.5 (± 5.3)***	***75.5 (± 5.1)***	***0.05***
***Mean arterial pressure***	***92.5 (± 6.6)***	***89.6 (± 5.6)***	***0.09***
Pre-nephrectomy renal function (mean, ± SD)	*** ***	*** ***	*** ***
***Creatinine mg/dL***	***0.87 (± 0.25)***	***0.85 (± 0.14)***	***0.3***
***GFR (CKD-EPI 2021)***	***96.8 (± 20.7)***	***107.8 (± 10.7)***	***0.02***
***Albuminuria mg/24 hours***	***12.6 (± 5.7)***	***5.3 (± 2.4)***	***0.0002***
***Proteinuria mg/24 hours***	***67.3 (± 43.2)***	***37.5 (± 13.4)***	***< 0.0001***
Renal function after 12 months (mean, ± SD)	*** ***	*** ***	*** ***
***Creatinine mg/dL***	***1.32 (± 0.26)***	***1.08 (± 0.16)***	***0.001***
***GFR (CKD-EPI 2021)***	***60.4 (± 8.9)***	***83 (± 12.06)***	***< 0.0001***
***Albuminuria mg/24 hours***	***14.2 (± 5.7)***	***8.2 (± 2.2)***	***0.0009***
***Proteinuria mg/24 hours***	***110.4 (± 28.6)***	***55.5 (± 12.1)***	***< 0.0001***
***Renal function CR*** (%, ± SD)	***60.8% (± 8.1)***	***76.3% (± 5.6)***	***< 0.0001***
TOTAL (n)	14	18	

SD: standard deviation. CR: compensation rate. GFR: glomerular filtration rate N.A.: non applicable. Source: The authors, 2023.

The risk factors tied to renal compensation of less than 70% 12 months after nephrectomy were analyzed using logistic regression and odds ratios (Table 3). The following variables were observed as risk factors with statistical significance: obesity, albuminuria and proteinuria. Higher initial GFR acted as a protective factor.

**Table 3:** Risk factors for gfr compensation < 70% 12 months post-nephrectomy among living kidney donors from a tertiary hospital in the amazon, 2016 to 2022, brazil

Variable	Odds Ratio (IC 95%)	*p* value
Male gender	***0.8 (0.19–3.2)***	***0.96***
Obesity	***10.6 (1.7–65.2)***	0.01*
Initial GFR	***0.92 (0.85–0.99)***	0.01*
Albuminuria	***2.41 (1.2–4.84)***	< 0.0001*
Proteinuria	***1.14 (1.03–1.25)***	< 0.0001*
Age	***1.03 (0.95–1.12)***	***0.35***
Mean arterial pressure	***1.08 (0.96–1.23)***	***0.16***

* Statistically significant value. GFR: glomerular filtration rate. Source: The authors, 2023.

## Discussion

The living kidney donors included in this study about the risk factors of lower renal compensation after one year of nephrectomy were followed at the Lower Amazon Regional Hospital (HRBA), an important referral center for nephrology care in Northern Brazil in the state of Pará. Since its establishment in 2016, more living donor kidney transplants were performed in the HRBA than in any of the seven transplant centers in Northern Brazil. Currently, 38% of all living kidney donor transplants performed in the region are carried out in the HRBA^18^. Donor follow-up is carried out in multiple stages after nephrectomy, with visits 15 and 30 days after the procedure, followed by biannual visits in the first year and annual visits thereafter, with 100% adherence among donors registered at the center. The collected data permits the analysis of donor epidemiological profiles, comorbidities and kidney function before and after nephrectomy.

Donor data also allow the analysis of factors potentially associated with a lower rate of renal compensation. Renal compensation may directly affect the selection of potential donors, as well as long-term follow-up protocol. Patients with a single kidney, whether due to congenital causes, trauma or nephrectomy for donation, develop means for compensating the loss of glomerular filtration rate via hyperfiltration, as described in animal models^19^. Special attention is required for living kidney donors given their chances of developing CKD and end-stage renal disease.

The donors in our population were predominantly males, although females were not substantially outnumbered. Other studies analyzing kidney donors found that the majority of living kidney donors were females, while recipients were mostly males^20^. Some factors seem to be involved in these findings, such as the expectation of altruism in women, in addition to the higher incidence of comorbidities, such as hypertension and diabetes, in men, making them less eligible for a possible donation^20^. Nevertheless, in our center there seemed to be a more balanced distribution of donors between the two sexes, which may signal a cultural difference or possibly a difference in the selection criteria adopted in Brazil versus other countries in which studies have been carried out.

Male gender has been considered a possible risk factor for lower rates of renal compensation within a year of nephrectomy one year, although not universally. According to a meta-analysis by Bellini et al.^21^, sex had no impact on the GFR one year after nephrectomy^21^. Nevertheless, a higher incidence of end-stage renal disease was described in male donors and longer short- and long-term survival among female donors. Kim et al.^22^ found that insufficient renal recovery was more common in men, with an increased risk of 44% compared to women, while Kim et al.^23^ found that females had higher GFR after nephrectomy than males. Interestingly, in the long term, estrogen has antioxidant and nephroprotective functions, which may explain the lower incidence of CKD among female donors, although the relationship between gender and post-donation kidney function has not been completely understood^24^.

As seen in the literature, donor mean age was 40.9 (± 8.8) years in our study. According to a recent meta-analysis, the estimated mean GFR in donors over 60 years of age was 9.54 mL/min/1.73m^2^ lower than the GRF of younger donors^21^. Nevertheless, no significant differences were found in serum creatinine, GFR a year after nephrectomy, proteinuria or survival between older and younger kidney donors^21^. On the other hand, Kim et al.^22^ reported lower levels of renal compensation in older donors, with increases of one year in age associated with increased risk of insufficient renal recovery by 3%. Kim et al.^23^ studied donor aging and showed that at each year the GFR decreased by 0.6 mL/min/1.73 m^2^.

In our study, donor age did not affect renal compensation rates. Donor aging, however, seems to have a greater impact on long-term follow-up, especially due to the appearance of common comorbidities such as diabetes, hypertension, cardiovascular disease and metabolic syndromes^25^. Adequate follow-up after donation plays a key role in preventing complications and constitutes a challenge for transplant centers given the low adherence of most donors over longer periods of time^26^.

Donor kidney function is one of the central elements in the selection of viable candidates for donation. The main objective of donor evaluation is to ensure the safety and well-being of prospective donors and determine that the risks to the donor are acceptable. In our study, we used the variables creatinine, GFR, albuminuria and proteinuria as parameters to evaluate the kidney function of the population. The manual published by the ABTO lists proteinuria > 250 mg/24 h and/or microalbuminuria > 30 mg/24 h as absolute contraindications for donation, since they constitute evidence of kidney disease, in addition to a GFR < 90 mL /min/1.73 m^2 27^. Individuals deemed healthy based on kidney function parameters are thus selected to minimize the chances of kidney disease developing in the long term. According to the Kidney Disease: Improving Global Outcomes (KDIGO) initiative, patients with a GFR between 89 and 60 mL/min/1.73m^2^ must be assessed individually and may become eligible donors depending on other health parameters^15^.

In our population, all patients had a GFR > 90 mL/min/1.73m^2^ before donation. However, when the GFR was analyzed as a risk factor for inadequate renal compensation after nephrectomy, we found that higher levels of GFR acted as a protective factor against having a renal compensation rate < 70%. In this sense, our study indicated the existence of a possible association between lower GFR values and lower rates of renal compensation, even though all individuals in our study population had normal parameters before nephrectomy. Kim et al.^22^ found several factors associated with a lower rate of renal compensation (GFR < 60 mL/min/1.73m^2^), including having a lower GFR before donation and a greater change in the baseline GFR in the first month after donation. The authors estimated that for every 1 mL/min/1.73m^2^ increase in pre-donation GFR, the risk of developing a GFR < 60 mL/min/1.73m^2^ decreased by 10%^22^. Other studies that evaluated factors associated with the onset of CKD in kidney donors also found that a higher GFR before donation was a protective factor against the development of CKD^23,24^. On the other hand, Massie et al.^27^ did not find a significant association between pre-donation GFR and end-stage renal disease, even though GFR levels six months after donation were a significant marker.

One year after nephrectomy, 18 of the 32 patients reached an average GFR of 83 mL/min/1.73m^2^, which reflects a normal decrease in GFR. Considering that the removal of a kidney results in the loss of approximately 50% of renal function in humans with subsequent compensation of the GFR to approximately 70%, most patients were able to achieve adequate compensation. The other 14 patients reached an average GFR of 60.4 mL/min/1.73m^2^, a borderline level for kidney function deterioration. This is an important factor in the analysis and monitoring of donors, since decreases in the GFR have been associated with increases in cardiovascular mortality and end-stage renal disease^28^. An interesting element described in prospective studies with longer follow-up times, such as the one by Kasiske et al.^29^, is the tendency for the GFR in living kidney donors to decrease in the first six months after nephrectomy when compared to that of controls, a trend that is reversed in the long term, with the GFR becoming stable in living kidney donors and decreasing in controls.

Pre-nephrectomy GFR, proteinuria and albuminuria were within the ranges recommended by the KDIGO and the ABTO for the selection of donors^15,30^. However, the latter two were related to a greater risk of poor renal compensation, demonstrating that even apparently healthy donors may be more prone to developing lower renal compensation rates with increases in proteinuria and albuminuria. Both the group with adequate renal compensation and the group that failed to reach this standard did not attain levels of albuminuria that might indicate kidney disease. Patients with a higher albumin/creatinine or protein/creatinine ratio in urine are at greater risk of developing end-stage renal disease, according to risk calculators developed for the North American population^31,32^.

The most widely evaluated comorbidities present in living kidney donors are obesity and hypertension. In our study, obesity was a risk factor for lower renal compensation. Although there is no strict guideline in most countries prohibiting donors with these conditions, most candidates with a BMI > 35-40 kg/m^2^ are rejected due to concerns about long-term deterioration of kidney function^33^. According to the ABTO, individuals with a BMI > 35 kg/m^2^ must not be accepted as donors in Brazil and individuals with a BMI between 30 and 35 kg/m2 must be individually evaluated and advised to implement lifestyle changes^30^. In fact, obesity impacts kidney health through several mechanisms, including obesity-related glomerulopathy, and has been associated with the development of CKD^33^.

Studies with long-term donor follow-up demonstrated that obese donors faced a greater decline in GFR, in addition to a higher incidence of diabetes mellitus and hypertension^33,34^. Other authors described a higher risk of end-stage renal disease in obese donors^35^. Donors with a BMI > 30 kg/m2 had an average GFR 2.70 mL/min/1.73 m^2^ lower than donors with a BMI < 30 kg/m^2^, in addition to having significantly higher proteinuria one year after donation^21^. Recent evidence generally corroborates our findings related to obesity.

We did not find risk factors for lower renal compensation associated with other comorbid conditions. This is probably due to the low frequency of other conditions in our population and the ABTO exclusion criteria, which greatly limits the acceptance of hypertensive individuals and completely excludes individuals with diabetes. Hypertension is a well-studied risk factor for CKD in living kidney donors. In a Korean study enrolling 456 living kidney donors, higher systolic blood pressure and a history of hypertension were associated with increased risk of CKD. The same was found in an analysis including African donors, in which hypertension was a risk factor for lower GFR after donation^23^. Smoking has also been associated with higher risk of developing CKD due to the accumulation of risk factors, including cardiovascular risk for overweight and obesity^28,32,36^.

In general, the main concern that accompanies the evaluation of living kidney donors is the development of CKD and end-stage renal disease. Our results are in line with scientific literature and highlight points that may be critical for the selection and long-term monitoring of donors. It is clear that the period encompassed by the first six months to one year after donation is critical in the monitoring of donors, since this is when certain factors may appear and potentially cause the future development of CKD. Our study brings relevant contributions to the analysis of the risk factors for lower renal compensation during this time period, in addition to a more refined perspective over some variables currently used for the selection of donors and whether or not variables such as BMI, GFR, proteinuria and albuminuria might be considered using broader ranges.

The main limitations of this study are the fact that it was carried out at a single center and that it enrolled a small population. The low frequency of patients with comorbidities such as hypertension, smoking and alcoholism did not make it possible to adequately assess the impacts of these factors on renal compensation. Another limitation of our study was the use of the GFR estimated based on the CKD-EPI 2021 equation, which may underestimate the GFR, especially among individuals with a family history of CKD and blood pressure and albuminuria levels above normal limits, as pointed out by Luján et al.^37^.

## Conclusion

Obesity and increased levels of albuminuria and proteinuria negatively affect the rate of renal compensation one year after nephrectomy, while higher GFR levels play a protective role. The identification of these factors may increase the safety of the process used to select living kidney donors and enable the individualized monitoring of patients at higher risk. Carrying out large population studies is essential to better define the short- and long-term impacts of clinical variables on kidney function after nephrectomy.
