# Supplementary material for: Risk factors for lower renal compensation after nephrectomy: an analysis of living kidney donors in an Amazonian cohort
Source: J Bras Nefrol. 2024 Feb 9;46(3):e20230134. doi: 10.1590/2175-8239-JBN-2023-0134en (PMC11287976; doi:10.1590/2175-8239-JBN-2023-0134en)
Supplement: Translation

# Translation: Fatores de risco para menor compensação renal pós-nefrectomia: análise de doadores de rim vivo em uma coorte amazônica

**DOI:** 10.1590/2175-8239-JBN-2023-0134pt

**Authors:** Luan Moraes Ferreira, Gisela Gomes Batista, Leoneide Érica Maduro Bouillet, Emanuel Pinheiro Esposito

**Affiliations:** 1 Universidade do Estado do Pará, Faculdade de Medicina, Santarém, PA, Brasil.

**Keywords:** Transplante de Rim, Doadores Vivos, Obesidade, Albuminúria, Proteinúria

## Abstract

**Introdução:**

O transplante de rim de doador vivo é considerado a terapia renal substitutiva ideal por oferecer menor taxa de complicações e possibilitar uma resposta eficiente à grande demanda por enxertos no sistema de saúde. A seleção criteriosa e o acompanhamento adequado dos doadores constituem um pilar fundamental dessa modalidade terapêutica, sendo essencial a identificação dos indivíduos em maior risco de disfunção renal pós-nefrectomia.

**Objetivo:**

Identificar fatores de risco para uma Taxa de Compensação (TC) da função renal inferior a 70% 12 meses após a nefrectomia.

**Métodos:**

Estudo observacional, retrospectivo e longitudinal conduzido com doadores de rim vivo acompanhados no Hospital Regional do Baixo Amazonas entre 2016 e 2022. Foram coletados dados correspondentes a variáveis sociodemográficas, comorbidades e parâmetros de função renal.

**Resultados:**

Foram incluídos 32 pacientes na amostra final. Destes, 14 (43,75%) obtiveram TC < 70% 12 meses após a doação. A regressão logística identificou a obesidade (Odds Ratio [IC95%]: 10.6 [1.7–65.2]), albuminúria (Odds Ratio [IC95%]: 2.41 [1.2–4.84]) e proteinúria (Odds Ratio [IC95%]: 1.14 [1.03–1.25]) como fatores de risco. A taxa de filtração glomerular atuou como fator de proteção (Odds Ratio [IC95%]: 0.92 [0.85–0.99]).

**Conclusão:**

Obesidade, albuminúria e proteinúria demonstraram impacto negativo na taxa de compensação renal em curto prazo, o que reitera a necessidade de estudos acerca das implicações prognósticas desses fatores. Além disso, reforça-se a necessidade de avaliação cuidadosa e individualizada dos possíveis doadores, com acompanhamento rigoroso, especialmente para indivíduos de maior risco.

## Introdução

A Doença Renal Crônica (DRC) é uma das principais causas de mortalidade da sociedade moderna e acomete mais de 10% da população mundial, com aproximadamente 840 milhões de indivíduos atingidos, compondo um importante desafio à saúde pública^1^. É definida pela presença de anormalidades estruturais ou de função renal reduzida/dano renal por um período superior a 3 meses, e sua classificação é realizada a partir da Taxa de Filtração Glomerular (TFG) e albuminúria, sendo o estágio terminal – onde está indicada a Terapia Renal Substitutiva (TRS) – correspondente a uma TFG < 15 mL/min/1,73 m^2 2^. A TRS consiste em um amplo arsenal terapêutico, todavia o transplante a partir de doador vivo é considerado a terapia ideal, pois apresenta menores taxas de perda de enxerto e maior tempo de sobrevida quando comparado à doação a partir de indivíduo falecido, além de reduzir significativamente as limitações de vida diária impostas por modalidades como diálise peritoneal e hemodiálise^3,4^.

De acordo com a Organização Mundial de Saúde (OMS) e a Associação Brasileira de Transplante de Órgãos (ABTO), em 2022 o Brasil foi o quarto maior centro de transplante de rim em números absolutos no mundo, realizando 5.306 procedimentos em 2022 – atrás apenas dos Estados Unidos, da China e da Índia. Aproximadamente 14% deles (n = 733) correspondentes a enxertos originados de doadores vivos, com expectativas de crescimento nos próximos anos^5,6^.

Após a doação de rim, o indivíduo perde aproximadamente 50% da massa renal, o que leva invariavelmente a uma redução da TFG no período pós-transplante^7^. É esperado que o rim remanescente consiga compensar essa perda a partir de um processo de hiperfiltração glomerular, levando a uma recuperação de aproximadamente 70% da TFG basal após um ano^8–10^.

Embora inicialmente se acreditasse que o risco de disfunção renal pós-nefrectomia não fosse significativo para os doadores de rim vivo – com a hipótese de que fosse na verdade mais baixo que o da população em geral em virtude da seleção de indivíduos mais saudáveis –, estudos com melhor pareamento de grupos demonstraram um pequeno, porém estatisticamente significante, risco de DRC terminal em longo prazo nessa população^11–13^.

Pesquisas recentes dedicaram-se a analisar a função renal de doadores em curto prazo. Um estudo realizado em 2018 na Espanha identificou diferenças estatisticamente significantes nas taxas de compensação da função renal entre doadores de rim vivo após um ano de seguimento^7^. Em análise realizada em 2020 na Coreia do Sul, a obesidade e o Índice de Massa Corpórea (IMC) foram associados a decréscimos mais acentuados da TFG pós-nefrectomia^14^. Contudo, a presente literatura científica carece de mais evidências acerca da identificação de fatores capazes de influenciar a recuperação da função renal em curto prazo após a doação, o que poderia auxiliar na identificação de indivíduos em maior risco de desenvolver DRC.

O processo de seleção de doadores de rim vivo tem como um de seus objetivos centrais a minimização de riscos ao doador, incorporando múltiplas etapas de segurança e fornecimento de informações, sendo essencial a constante atualização da literatura científica acerca dos fatores associados a um pior prognóstico pós-doação em curto e longo prazo, aumentando a rede de segurança em torno de potenciais doadores e permitindo um seguimento individualizado dos indivíduos em maior risco^15^. O objetivo deste trabalho foi identificar fatores de risco associados a uma taxa de compensação da função renal < 70% um ano após a nefrectomia.

## Métodos

Tratou-se de um estudo de desenho observacional, retrospectivo e longitudinal compreendendo uma coorte de pacientes de ambos os sexos e em diversas faixas etárias que realizaram nefrectomia para a doação de rim em um hospital terciário localizado na Amazônia Central no período de janeiro de 2016 a dezembro de 2022. Foram excluídos os pacientes que não realizaram a consulta de seguimento ambulatorial 12 meses após o transplante renal ou que não haviam completado um ano da cirurgia de nefrectomia até a data da coleta de dados.

O processo de aplicação dos critérios de inclusão e exclusão para a composição da amostra final da pesquisa está descrito na Figura 1.

**Figura 1.** Fluxograma de aplicação dos critérios de inclusão e exclusão.

As variáveis demográficas incluídas para a análise foram: sexo, idade e raça/cor. Foram analisadas as seguintes variáveis de interesse clínico: comorbidades (obesidade – definida por IMC ≥ 30 kg/m^2^, conforme recomendação do *National Institute for Health and Care Excelence* –, hipertensão arterial sistêmica, dislipidemia, alcoolismo e tabagismo); pressão arterial e parâmetros de função renal (creatinina, TFG, albuminúria e proteinúria)^16^. As medidas de albuminúria e proteinúria foram obtidas a partir da coleta de urina de 24 horas. Para a estimativa da TFG a partir da creatinina sérica, foi utilizada a fórmula CKD-EPI 2021^17^. Foi tomado como referência para a estimativa da TFG pré-transplante o nível de creatinina sérica na última consulta ambulatorial pré-doação. Para o cálculo da TFG 12 meses após a nefrectomia, foi considerado o nível de creatinina na consulta de seguimento um ano após o procedimento. Em nosso estudo, não levamos em consideração depuração de creatinina endógena pelo fato de esse parâmetro não fazer parte dos exames de rotina para os doadores de rim do centro escolhido.

A coleta de dados nos prontuários dos pacientes incluídos foi realizada no período de 1 a 15 abril de 2023, considerando os dados da última consulta pré-transplante renal e da consulta de seguimento ambulatorial após um ano da doação. Obteve-se a taxa de compensação da função renal a partir da TFG 12 meses pós-nefrectomia e da TFG pré-doação, conforme método proposto por Burballa *et al*.^7^: (Taxa de compensação renal):(TFG 1 ano pós-nefrectomia/TFG pré-nefrectomia)*100.

Considerou-se como ponto de corte de normalidade uma taxa de compensação da função renal em 12 meses ≥ 70%, conforme estudos prévios realizados em doadores de rim vivo^7–10^.

Para a análise descritiva foram utilizadas frequência absoluta e relativa para variáveis categóricas, e média e desvio-padrão para variáveis quantitativas com distribuição normal, e mediana e intervalo interquartil para aquelas de distribuição não normal. A normalidade da amostra foi verificada pelo teste de Shapiro-Wilk. Para a comparação entre variáveis categóricas, foi utilizado o teste qui-quadrado, enquanto para as variáveis numéricas foi utilizado o teste T. Também foi realizada a verificação de possíveis fatores de risco para uma taxa de compensação < 70% a partir de regressão logística, utilizando *odds ratio* e um intervalo de confiança (IC) de 95%. Todos os testes foram realizados no software Stata, versão 14.0. Para todos os testes estatísticos foi considerado como significante *p* < 0,05.

O estudo foi realizado em conformidade com a Resolução 466/2012 do Conselho Nacional de Saúde, obtendo aprovação do Comitê de Ética em Pesquisa da Universidade do Estado do Pará para a sua realização (CAAE: 67526923.0.0000.5168/ Número do Parecer: 5.719.668). Consentimento informado foi obtido de todos os participantes.

## Resultados

Foram incluídos 32 participantes para a análise final. Predominaram indivíduos do sexo masculino (n = 17, 53,2%), da raça/cor parda (n = 29, 90,6%), com idade média de 40,9 ± 8,8 anos. No que tange aos parâmetros clínicos, a comorbidade predominante foi a obesidade (n = 10, 31,2%). A pressão arterial sistólica e diastólica observadas na amostra foram respectivamente 119,6 (± 9,6) e 76,7 (± 5,4), com pressão arterial média de 90,9 (± 6,1). Quanto à função renal, observou-se incremento da creatinina ao longo do primeiro ano pós-nefrectomia (de 0,86 ± 0,14 para 1,2 ± 0,24), bem como redução da TFG (de 104 ± 12,3 para 69,8 ± 16,8). Com relação à albuminúria, obteve-se aumento de 8,5 ± 5,5 mg para 10,8 ± 5 mg em 12 meses, também houve aumento na proteinúria de 58,3 ± 31,9 mg para 79,5 ± 33,7 mg. Os parâmetros sociodemográficos e clínicos pré-doação e a função renal 12 meses pós-nefrectomia da população analisada no estudo estão descritos na Tabela 1.

**Tabela 1:** Caracterização sociodemográfica e clínica dos doadores de rim vivo em hospital terciário na amazônia, de 2016 a 2022, brasil

Variável	Frequência
Sexo (n, %)	
***Masculino***	*17 (53.2)*
***Feminino***	*15 (46,8)*
Idade (média, ± DP)	*40.9 (± 8.8)*
Raça/cor (n, %)	
***Branco***	*2 (6.2)*
***Pardo***	*29 (90.6)*
***Preto***	*1 (3.1)*
Comorbidades (n, %)	
***Obesidade***	*10 (31.2)*
***Hipertensão arterial sistêmica***	*3 (9.3)*
***Dislipidemia***	*1 (3.1)*
***Tabagismo***	*5 (15.6)*
***Alcoolismo***	*5 (15.6)*
Pressão arterial (média, ± DP)	* *
***Pressão arterial sistólica***	*119.6 (± 9.6)*
***Pressão arterial diastólica***	*76.7 (± 5.4)*
***Pressão arterial média***	*90.9 (± 6.1)*
Função renal pré-nefrectomia (média, ± DP)	
***Creatinina mg/dL***	*0.86 (± 0.14)*
***TFG (CKD-EPI 2021)***	*104 (± 12.3)*
***Albuminúria mg/24 horas***	*8.5 (± 5.5)*
***Proteinúria mg/24 horas***	*58.3 (± 31.9)*
Função renal após 12 meses (média, ± DP)	
***Creatinina mg/dL***	*1.2 (± 0.24)*
***TFG (CKD-EPI 2021)***	*69.8 (± 16.8)*
***Albuminúria mg/24 horas***	*10.8 (± 5)*
***Proteinúria mg/24 horas***	*79.5 (± 33.7)*
***TC da função renal*** (%, ±)	*69.5% (± 10.3)*
TOTAL (n)	*32*

DP: desvio-padrão. TC: Taxa de compensação. TFG: taxa de filtração glomerular. Fonte: Elaborado pelos autores, 2023.

Os 32 indivíduos incluídos no estudo foram divididos em dois grupos a partir da taxa de compensação da função renal após um ano da nefrectomia, correspondendo o Grupo α a uma taxa de compensação < 70% e o Grupo β a uma compensação > 70%. As diferenças consideradas estatisticamente significantes foram observadas nas seguintes variáveis pré-doação: obesidade, TFG, albuminúria e proteinúria. Foi encontrada significância estatística em todas as variáveis de função renal após 12 meses da nefrectomia. Os dados comparativos entre os grupos supracitados estão descritos na Tabela 2.

**Tabela 2:** Diferença entre os grupos referente à taxa de compensação renal um ano após a nefrectomia em um hospital terciário na amazônia, de 2016 a 2022, brasil

Variável	Total		
	Grupo α – TC da função renal < 70%	Grupo β – TC da função renal > 70%	*p* valor
Sexo (n, %)	* *	* *	* *
***Masculino***	*7 (50)*	*10 (55.5)*	*0.95*
***Feminino***	*7 (50)*	*8 (44.4)*	
Idade (média, ± DP)	*42.5 (± 9.2)*	*39.7 (± 8.4)*	*0.18*
Raça/cor (n, %)	* *	* *	* *
***Branco***	*1 (7.1)*	*1 (5.5)*	*0.98*
***Pardo***	*12 (85.7)*	*17 (94.4)*	
***Preto***	*1 (7.1)*	*0*	
Comorbidades (n, %)	* *	* *	* *
***Obesidade***	*8 (57.1)*	*2 (11.1)*	*0.005*
***Hipertensão arterial sistêmica***	*2 (14.2)*	*1 (5.5)*	*0.4*
***Dislipidemia***	*1 (4.1)*	*0*	*N.A.*
***Tabagismo***	*3 (21.4)*	*2 (11.1)*	*0.3*
***Alcoolismo***	*3 (21.4)*	*2 (11.1)*	*0.3*
Pressão arterial (média, ± DP)	* *	* *	* *
***Pressão arterial sistólica***	*120.7 (± 9.9)*	*118 (± 9.6)*	*0.3*
***Pressão arterial diastólica***	*78.5 (± 5.3)*	*75.5 (± 5.1)*	*0.05*
***Pressão arterial média***	*92.5 (± 6.6)*	*89.6 (± 5.6)*	*0.09*
Função renal pré-nefrectomia (média, ± DP)	* *	* *	* *
***Creatinina mg/dL***	*0.87 (± 0.25)*	*0.85 (± 0.14)*	*0.3*
***TFG (CKD-EPI 2021)***	*96.8 (± 20.7)*	*107.8 (± 10.7)*	*0.02*
***Albuminúria mg/24 horas***	*12.6 (± 5.7)*	*5.3 (± 2.4)*	*0.0002*
***Proteinúria mg/24 horas***	*67.3 (± 43.2)*	*37.5 (± 13.4)*	*< 0.0001*
Função renal após um ano (média, ± DP)	* *	* *	* *
***Creatinina mg/dL***	*1.32 (± 0.26)*	*1.08 (± 0.16)*	*0.001*
***TFG (CKD-EPI 2021)***	*60.4 (± 8.9)*	*83 (± 12.06)*	*< 0.0001*
***Albuminúria mg/ 24 horas***	*14.2 (± 5.7)*	*8.2 (± 2.2)*	*0.0009*
***Proteinúria mg/ 24 horas***	*110.4 (± 28.6)*	*55.5 (± 12.1)*	*< 0.0001*
***Taxa de compensação renal*** (%, ±)	*60.8% (± 8.1)*	*76.3% (± 5.6)*	*< 0.0001*
Total (n)	14	18	

DP: desvio-padrão. TC: taxa de compensação. TFG: taxa de filtração glomerular. N.A.: não aplicável. Fonte: Elaborado pelos autores, 2023.

Foi realizado o levantamento de fatores de risco para uma compensação inferior a 70% em 12 meses a partir de regressão logística e *odds ratio* (Tabela 3). Dentre as variáveis testadas, foram observados como fatores de risco com significância estatística as variáveis: obesidade, albuminúria e proteinúria. Notou-se também que valores mais altos de TFG inicial atuaram como fator de proteção.

**Tabela 3:** Fatores de risco para uma compensação da tfg < 70% 12 meses pós-nefrectomia entre doadores de rim vivo de um hospital terciário na amazônia, de 2016 a 2022, brasil

Variável	Odds Ratio (IC 95%)	p Valor
Sexo masculino	*0.8 (0.19–3.2)*	*0.96*
Obesidade	*10.6 (1.7–65.2)*	0.01*
TFG inicial	*0.92 (0.85–0.99)*	0.01*
Albuminúria	*2.41 (1.2–4.84)*	< 0.0001*
Proteinúria	*1.14 (1.03–1.25)*	< 0.0001*
Idade	*1.03 (0.95–1.12)*	*0.35*
Pressão arterial média	*1.08 (0.96–1.23)*	*0.16*

* Valor estatisticamente significante. TFG: taxa de filtração glomerular. Fonte: Elaborado pelos autores, 2023.

## Discussão

Os doadores de rim vivo participantes deste estudo sobre os fatores de risco para uma menor compensação renal após um ano de nefrectomia são acompanhados pelo Hospital Regional do Baixo Amazonas (HRBA), importante centro de referência para nefrologia na região Norte do Brasil e no estado do Pará. Dentre os 7 centros de transplante nortistas existentes, o HRBA é o que mais realiza transplantes de rim na modalidade de doador vivo desde sua implantação, em 2016, sendo responsável por 38% dos procedimentos desse tipo na região^18^. O acompanhamento desses pacientes após a doação é feito em múltiplas etapas após a doação, com consultas 15 e 30 dias após a nefrectomia, prosseguindo para encontros semestrais no primeiro ano e a partir disso anualmente, com 100% de aderência entre os doadores registrados no estabelecimento. A partir disso, ao analisar os dados encontrados foi possível identificar o perfil epidemiológico, comorbidades e função renal desses pacientes pré e pós-nefrectomia.

Esses dados possibilitam a análise de fatores que podem estar associados a uma menor taxa de compensação renal nesses pacientes. A compensação renal é um fator que pode influenciar diretamente a seleção dos possíveis doadores, bem como seu acompanhamento em longo prazo. Sabe-se que pacientes com rim único, seja por causas congênitas, traumas ou nefrectomia para doação, possuem mecanismos de compensação da taxa de filtração glomerular associados à hiperfiltração, conforme descrito em modelos animais^19^. Há uma atenção especial voltada aos doadores de rim vivo e principalmente à possibilidade do desenvolvimento de DRC e ainda estágio terminal de doença renal nesses pacientes.

A maior parte dos doadores em nossa amostra é do sexo masculino, mas o número não se distancia muito do sexo feminino. Alguns estudos que também analisaram o perfil dos pacientes doadores demonstraram que a maior parte das doações de rim vivo provém do sexo feminino, enquanto o número de receptores é majoritariamente do sexo masculino^20^. Alguns fatores parecem estar envolvidos nesses achados, como a expectativa de altruísmo sobre as mulheres, além da maior incidência de comorbidades, como hipertensão e diabetes em homens, tornando-os menos elegíveis para uma possível doação^20^. Ainda assim, no centro analisado parece haver uma equiparação entre os dois sexos, o que pode sinalizar uma diferença cultural ou mesmo dos critérios de seleção entre o Brasil e outros países onde tais pesquisas foram feitas.

O sexo masculino foi analisado como possível fator de risco para uma menor taxa de compensação renal em um ano, mas não figurou como tal. De acordo com metanálise de Bellini *et al*.^21^, o sexo não teve influência sobre a TFG um ano após a nefrectomia^21^. Apesar disso, foi demonstrada maior incidência de doença renal terminal nos doadores do sexo masculino, além de maior sobrevida em curto e longo prazo nas doadoras do sexo feminino. No estudo de Kim *et al*.^22^, uma recuperação renal insuficiente foi mais comum em homens, com risco aumentado em 44% comparado às mulheres, e no estudo de Kim *et al*.^23^, as mulheres apresentaram TFG pós-nefrectomia maior do que a dos homens. É apontado que, em longo prazo, o estrogênio possua funções antioxidantes e nefroprotetoras, o que pode explicar a menor incidência de DRC entre doadores do sexo feminino, embora a relação do gênero com a função renal pós-doação ainda não seja completamente compreendida^24^.

Quanto à idade, foi encontrada uma média de 40,9 (± 8,8) anos, o que também é caracterizado em outros estudos como um achado comum. Segundo metanálise recente, a TFG estimada em doadores com idade superior a 60 anos foi em média 9,54 mL/min/1,73m^2^ menor do que a de doadores mais jovens^21^. Apesar disso, não foram encontradas diferenças significativas na creatinina sérica, TGF um ano pós-nefrectomia, proteinúria e sobrevida de pacientes doadores de rim mais velhos e mais jovens^21^. Por outro lado, no estudo de Kim *et al*.^22^ houve menor compensação renal nos doadores mais velhos, em que cada 1 ano na idade aumentou o risco de recuperação renal insuficiente em 3%, assim como no estudo de Kim *et al*.^23^, conforme o qual a idade mostrou um efeito de 0,6 mL/min/1,73 m^2^ na diminuição da TFG por incremento de 1 ano.

Em nosso estudo, a idade dos pacientes não se configurou como fator que influenciasse as taxas de compensação renal. A diferença de idade de um paciente doador parece ter maior influência no seu acompanhamento em longo prazo, principalmente quando pensamos no aparecimento de comorbidades comuns ao avançar da idade, como diabetes, hipertensão, doenças cardiovasculares e síndromes metabólicas^25^. O acompanhamento adequado após a doação desempenha um papel essencial na prevenção de complicações e constitui um desafio aos centros de transplante tendo em vista a baixa aderência da maioria dos doadores em períodos de tempo maiores^26^.

A função renal do paciente doador é um dos pontos fundamentais para a seleção de candidatos viáveis e o principal objetivo da avaliação é garantir a segurança e o bem-estar do candidato e definir que os riscos para o doador sejam aceitáveis. Em nosso estudo, usamos como parâmetros para avaliação da função renal da amostra as variáveis creatinina, TFG, albuminúria e proteinúria. O manual de transplante da ABTO, para as variáveis analisadas, traz como contraindicações absolutas para a doação proteinúria > 250 mg/24 h e/ou microalbuminúria > 30 mg/24 h, o que indicaria evidência de doença renal, além de TFG < 90 mL/min/1,73 m^2 27^. Dessa forma, são selecionados aqueles em que os parâmetros de função renal correspondam a um indivíduo saudável, minimizando assim os riscos de desenvolvimento de doença renal em longo prazo. De acordo com o *Kidney Disease: Improving Global Outcomes* (KDIGO), pacientes com TFG que estejam entre 89 e 60 mL/min/1,73m^2^ devem ter sua avaliação individualizada e podem ser incluídos para doação dependendo de outros parâmetros de saúde^15^.

Em nossa amostra, todos os pacientes apresentavam TFG > 90 mL/min/1,73m^2^ antes da doação. Apesar disso, ao analisarmos a TFG como fator de risco para compensação renal inadequada após nefrectomia, foi demonstrado que valores mais altos atuaram como fator de proteção para uma TC < 70%. Nesse sentido, os resultados do nosso estudo indicam haver uma possível associação entre menores valores da TFG com menores taxas de compensação renal, ainda que todos os indivíduos da amostra estivessem dentro dos parâmetros de normalidade pré-nefrectomia. O estudo de Kim *et al*.^22^ encontrou diversos fatores associados a uma menor taxa de recuperação renal (TFG < 60 mL/min/1,73m^2^) e entre eles esteve uma menor TFG antes da doação, bem como maior alteração dessa TFG basal no primeiro mês após a doação. Foi estimado nesse estudo que a cada 1 mL/min/1,73m^2^ de aumento na TFG pré-doação o risco para o desenvolvimento de TFG < 60 mL/min/1,73m^2^ reduziu em 10%^22^. Outros estudos que avaliaram fatores associados ao aparecimento de DRC em pacientes doadores de rim também encontraram maior TFG antes da doação como fator de proteção para o desenvolvimento de DRC^23,24^. Por outro lado, no estudo de Massie *et al*.^27^ não foi encontrada associação significativa entre TFG pré-doação e doença renal terminal, ainda que os valores de TFG 6 meses pós-doação se apresentassem como um marcador significativo.

Após a retirada do rim, 18 dos 32 pacientes atingiram a média de 83 mL/min/1,73m^2^ após um ano, o que caracteriza uma diminuição normal da TFG. Considerando que a uninefrectomia resulta em perda de aproximadamente 50% da função renal em humanos com posterior compensação da TFG para aproximadamente 70%, a maior parte dos pacientes conseguiu atingir a compensação adequada. Os outros 14 pacientes atingiram a média de 60,4 mL/min/1,73m^2^, média limítrofe para estágios de deterioração da função renal. Esse é um fator importante na análise e no acompanhamento desses pacientes, uma vez que a diminuição da TFG estimada tem sido associada ao aumento da mortalidade cardiovascular e de doença renal terminal^28^. Um ponto de interesse que trazem alguns estudos prospectivos, com tempo de acompanhamento maior, como o de Kasiske *et al*.^29^, é a tendência de que a TFG dos doadores de rim vivo diminua nos primeiros seis meses quando comparada à de pacientes controle, mas que em longo prazo esse parâmetro se inverta, com estabilidade dos pacientes doadores de rim vivo e diminuição da TFG nos pacientes controle.

Assim como a TFG dos pacientes estava dentro da normalidade antes da doação, a proteinúria e albuminúria também estavam na faixa recomenda pelo KDIGO e pela ABTO para seleção desses candidatos^15,30^. Apesar disso, essas duas variáveis se relacionaram com maior risco para compensação renal inadequada, demonstrando que mesmo pacientes aparentemente saudáveis podem estar mais propensos a menor taxa de compensação conforme essas variáveis aumentam seus valores. Tanto o grupo com compensação renal adequada quanto o grupo que não atingiu essa faixa não chegaram a níveis de albuminúria que traduzissem doença renal. Pacientes com maior relação albumina/creatinina ou proteína/creatinina na urina apresentam maiores riscos de desenvolver doença renal terminal de acordo com calculadoras de risco desenvolvidas para a população norte-americana^31,32^.

Quanto às comorbidades presentes nos doadores de rim vivo, as mais amplamente avaliadas pelos estudos recentes são a obesidade e a hipertensão. Em nosso estudo foi possível observar a obesidade como fator de risco para menor compensação renal. Embora não haja uma diretriz rígida na maioria dos países que proíba a doação de indivíduos obesos, a maior parte dos candidatos com IMC > 35-40 kg/m^2^ é rejeitada devido a preocupações com a deterioração da função renal em longo prazo^33^. De acordo com a ABTO, pessoas com IMC > 35 kg/m^2^ não devem ser aceitas como doadoras no Brasil e pessoas com IMC entre 30 e 35 kg/m^2^ devem ser avaliadas individualmente e aconselhadas a mudar seu estilo de vida^30^. De fato, a obesidade tem impacto sobre a saúde renal por diversos mecanismos, dentre eles a glomerulopatia relacionada à obesidade, além de predispor ao desenvolvimento de DRC^33^.

Estudos com seguimento de longo prazo demonstraram que os doadores obesos enfrentaram maior declínio da TFG, além de maior incidência de diabete mellitus e hipertensão^33,34^. Enquanto isso, outro estudo demonstrou maior risco de doença renal terminal em doadores obesos^35^. Doadores com IMC > 30 kg/m^2^ tiveram TFG em média 2,70 mL/min/1,73m^2^ menor do que a de doadores com IMC < 30 kg/m^2^, além do achado de proteinúria significativamente maior após um ano da doação^21^. Dessa forma, observamos que as evidências mais recentes em geral corroboram com nossos achados relacionados à obesidade.

Em relação às outras comorbidades, nosso estudo não encontrou fatores de risco para menor compensação renal. Esse fator provavelmente se deve à baixa frequência dessas condições em nossa amostra devido aos critérios de exclusão da ABTO, limitando bastante a aceitação de pacientes hipertensos e a exclusão total de pacientes diabéticos. Apesar disso, a hipertensão é bem estudada como fator de risco para DRC em doadores de rim vivo. Em um estudo coreano, no qual foram analisados 456 doadores vivos, a pressão sistólica mais alta e história de hipertensão estiveram associadas ao risco aumentado de DRC, assim como em análise feita com doadores africanos, na qual a hipertensão também figurou como fator de risco para diminuição da TFG após a doação^23^. O tabagismo também foi associado ao maior risco de desenvolvimento de DRC por fatores de risco acumulados, como risco cardiovascular por sobrepeso e obesidade em alguns estudos^28,32,36^.

De maneira geral, a principal preocupação que acompanha a avaliação dos doadores de rim vivo é o desenvolvimento de DRC ou mesmo de doença renal terminal. Nesse sentido, nossos resultados se alinham com a literatura científica e trazem destaque para pontos que podem ser críticos para a seleção e o acompanhamento desses pacientes em longo prazo. Percebe-se que o período crítico para acompanhamento desses pacientes se dá principalmente de seis meses a um ano, quando o aparecimento de determinados fatores pode influenciar o futuro desenvolvimento de DRC. Logo, nosso estudo traz contribuições relevantes ao analisar os fatores de risco para menor compensação renal justamente nesse período, além de um olhar mais refinado sobre algumas variáveis atualmente utilizadas para a seleção dos pacientes doadores e flexibilização ou não de alguns valores como IMC, TFG, proteinúria e albuminúria.

Este estudo tem como principais limitações ser unicêntrico e de pequeno tamanho amostral. A baixa frequência de pacientes com comorbidades como hipertensão, tabagismo e alcoolismo não possibilitou avaliar adequadamente os impactos desses fatores. Outra limitação de nosso estudo foi utilizar TFG estimada a partir do método CKD-EPI 2021, o que pode subestimar a TFG – especialmente entre indivíduos com história familiar de DRC e níveis pressóricos e de albuminúria acima dos limites de normalidade, conforme apontado por Luján *et al*.^37^.

## Conclusão

Obesidade e valores aumentados de albuminúria e de proteinúria afetam negativamente a taxa de compensação da função renal um ano após a nefrectomia, enquanto valores maiores de TFG desempenham papel protetor. A identificação desses fatores pode auxiliar no aumento da segurança no processo de seleção dos doadores de rim vivo, além de possibilitar individualização do acompanhamento de pacientes em maior risco. A realização de grandes estudos populacionais é fundamental para definir melhor os impactos em curto e longo prazo de variáveis clínicas na função renal após a nefrectomia.
